# Possible Right Coronary Artery Supply to Pericardial Adipose Tissue Adjacent to an Abnormal Proximal Ascending Aortic Wall: A Case Report

**DOI:** 10.7759/cureus.106078

**Published:** 2026-03-29

**Authors:** Hidekazu Takeuchi

**Affiliations:** 1 Internal Medicine (Cardiology), Takeuchi Naika Clinic, Ogachi-Gun, JPN

**Keywords:** apixaban, copd, fev1.0%, pericardial adipose tissue (pat), right coronary artery (rca), transesophageal echocardiography (tee)

## Abstract

A 63-year-old male patient with chronic obstructive pulmonary disease (COPD) was examined using cardiac computed tomography (CT) and transesophageal echocardiography (TEE) to estimate the presence of chronic thrombi, such as left atrial appendage (LAA) thrombi and pulmonary vein thrombi (PVTs). The patient presented with a strange thrombus-containing vessel over the anterior surface of the heart, which did not appear to be a coronary vein. The abnormal wall of the ascending aorta (AAo) was difficult to differentiate from the AAo thrombi, and a dark (low echogenic) mass was observed in the center of the abnormal AAo wall on TEE. The origin of the mass was unknown but could potentially be associated with atherosclerosis, a Valsalva aneurysm, aortic dissection, an aortic aneurysm, or aortic valve disease. The relationship between these factors and the coronary artery, as well as whether apixaban (a factor Xa inhibitor) could cure the cleft in the mass, remains unclear.

## Introduction

Previous studies have revealed that thrombi retrieved from patients with acute myocardial infarction (AMI), acute ischemic stroke (AIS), or pulmonary hypertension exhibit powder-like calcification and endothelialization [1] or contain fine and large calcifications [2], indicating that the thrombi were present for a long time period. I reported that most elderly patients with hypertension, dyslipidemia, chest pain, or diabetes have pulmonary vein thrombi (PVTs) [3,4] and that right lower pulmonary vein (RLPV) thrombi extend into the left atrium (LA) and are attached to the anterior wall of the LA [5]. Additionally, I reported the presence of a left atrial diverticulum around the attachment area [5]. Since 2025, transesophageal echocardiography (TEE) has revealed right middle pulmonary vein (RMPV) [6] or right upper pulmonary vein (RUPV) [7] thrombi connected to ascending aorta (AAo) thrombi through linear white thrombi suspected to be situated outside the heart and an abnormal right sidewall of the AAo; thus, differentiating AAo thrombi from the abnormal wall of the AAo is difficult [6-8].

AAo thrombi are floating thrombi that attach to erosive and atherosclerotic AAo walls [9-13] and sometimes cause AMI by obstructing the coronary artery. The floating thrombus generated on the atheromatous plaque in the AAo and immunostaining for the surface antigen CD34 revealed that CD34-positive endothelial cells were present on the erosion, along the stalk, and on the floating thrombus [11]. The mechanisms underlying the development of AAo thrombi are unclear, and methods for treating AAo thrombi have not been established. The linear white thrombi and AAo thrombi were partially resolved using warfarin and heparin [8] or apixaban [6]. In my previous reports, cardiac computed tomography (CT) did not reveal AAo thrombi or linear white thrombi, which are similar to left atrial thrombi that extend from PVTs [5-8]. Although these thrombi were partially resolved by apixaban, residual thrombi persisted during dose reduction [6]. Moreover, treatment with dabigatran and edoxaban for 22 months partially resolved the AAo thrombi and linear white thrombi and improved the cleft in the abnormal wall of the AAo [7]. However, whether apixaban can cure the cleft is unclear.

Epicardial adipose tissue (EAT) is associated with heart failure [14], atrial fibrillation, and coronary artery disease, and is supplied by branches of the coronary artery. EAT is primarily stored in the interventricular and atrioventricular grooves between the epicardium and the myocardium. Pericardial adipose tissue (PAT), on the other hand, is thought not to be supplied by the coronary artery and is associated with coronary artery calcification [15]. The present report clarifies that apixaban had partially resolved the condition, and CT suggested a possible atypical arterial branch from the right coronary artery (RCA) toward the PAT.

## Case presentation

A 63-year-old male patient with chronic obstructive pulmonary disease (COPD) was examined using cardiac CT and TEE to assess the presence of chronic thrombi, such as left atrial appendage (LAA) thrombi and PVTs. He had no symptoms of paralysis, chest pain, or exertional dyspnea in daily life. His blood pressure was 128/68 mmHg, his heart rate was 105 bpm, and his physical examination was unremarkable. His weight was 61 kg, and his height was 168 cm, with a BMI of 21.6 kg/m^2^. His brain natriuretic protein (BNP) concentration was 2.9 pg/mL (normal: <18.4 pg/mL), and his albumin-to-creatinine ratio (ACR) was 4.6 mg/gCr (normal: <30 mg/gCr). He had not been treated with warfarin or direct oral anticoagulants (DOACs) previously. His percentage forced expiratory volume in one second (FEV1.0%) was 60.2% (normal: >70%).

Electrocardiography (ECG) indicated a normal axis and sinus rhythm, no ST-T changes, and no notching. The serum D-dimer concentration was 0.7 μg/mL (normal: < 1.0 μg/mL), the protein S activity was 96% (normal: 74-132%), and the protein C activity was 135% (normal: 64-135%). The homocysteine concentration was 16.7 nmol/mL (normal: 5-15 nmol/mL).

**Figure 1:**
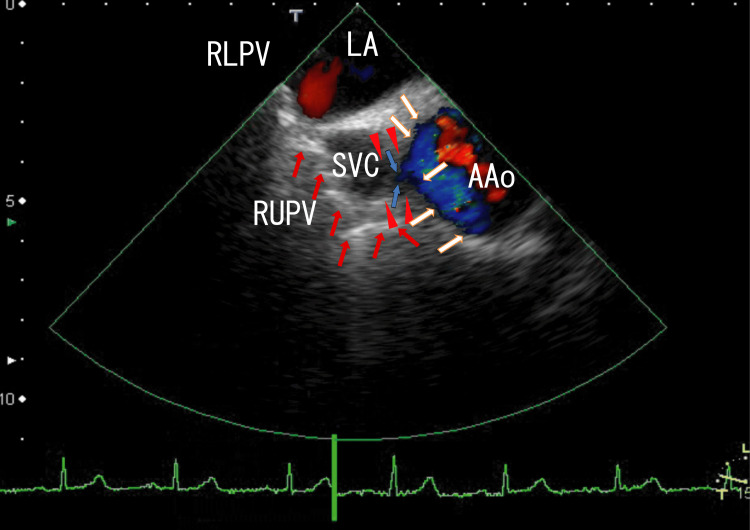
TEE image showing vessels with thrombi. TEE revealed linear vessels with white thrombi (red arrows) that connected RUPV thrombi to AAo thrombi, the latter of which were attached to the AAo wall (white arrows). The vessel was located beneath the SVC. Near this region, the structure of the AAo wall was abnormal, and a low-echogenic area was present (arrowheads). AAo thrombi were seen as blue areas within the AAo, indicating disturbed blood flow. Fine blue lines invading the AAo wall (blue arrows) indicated the presence of a cleft. TEE: transesophageal echocardiography; AAo: ascending aorta; RUPV: right upper pulmonary vein; SVC: superior vena cava; LA: left atrium; RLPV: right lower pulmonary vein

TEE revealed white (highly echogenic) thrombi around the ostia of the RUPV, which seemed to be connected to linear white thrombi situated on the anterior side of the superior vena cava (SVC) and reached white thrombi near the wall of the AAo (Figure 1, red arrows). There were rather vague whitish thrombi on the right side of the AAo wall (Figure 1, white arrows), the center of which was a dark area (low echogenic) (Figure 1, arrowheads). In the dark area on the AAo wall, there was a cleft, indicated with fine blue lines (Figure 1, blue arrows).

Video images revealed that the AAo thrombi moved with heartbeats; however, the RUPV and linear thrombi did not (Video 1), indicating that the linear white thrombi were not contained in the PAT and might have extended past the epicardium. Furthermore, the video images revealed the cleft more clearly.

**Video 1:** TEE video showing vessels with thrombi. TEE revealed a linear vessel with white thrombi that connected the RUPV thrombi to AAo thrombi, the latter of which were attached to the AAo wall. The vessel was located anterior to the SVC and did not move with heartbeats. The structure of the AAo wall was abnormal, with a low-echogenic area present at its center. AAo thrombi were seen as blurred white or blue areas within the AAo, indicating disturbed blood flow. Fine blue lines invading the AAo wall indicated the presence of a cleft. The angle of this video was the same as that in Figure 1. TEE: transesophageal echocardiography; AAo: ascending aorta; RUPV: right upper pulmonary vein; SVC: superior vena cava

**Figure 2:**
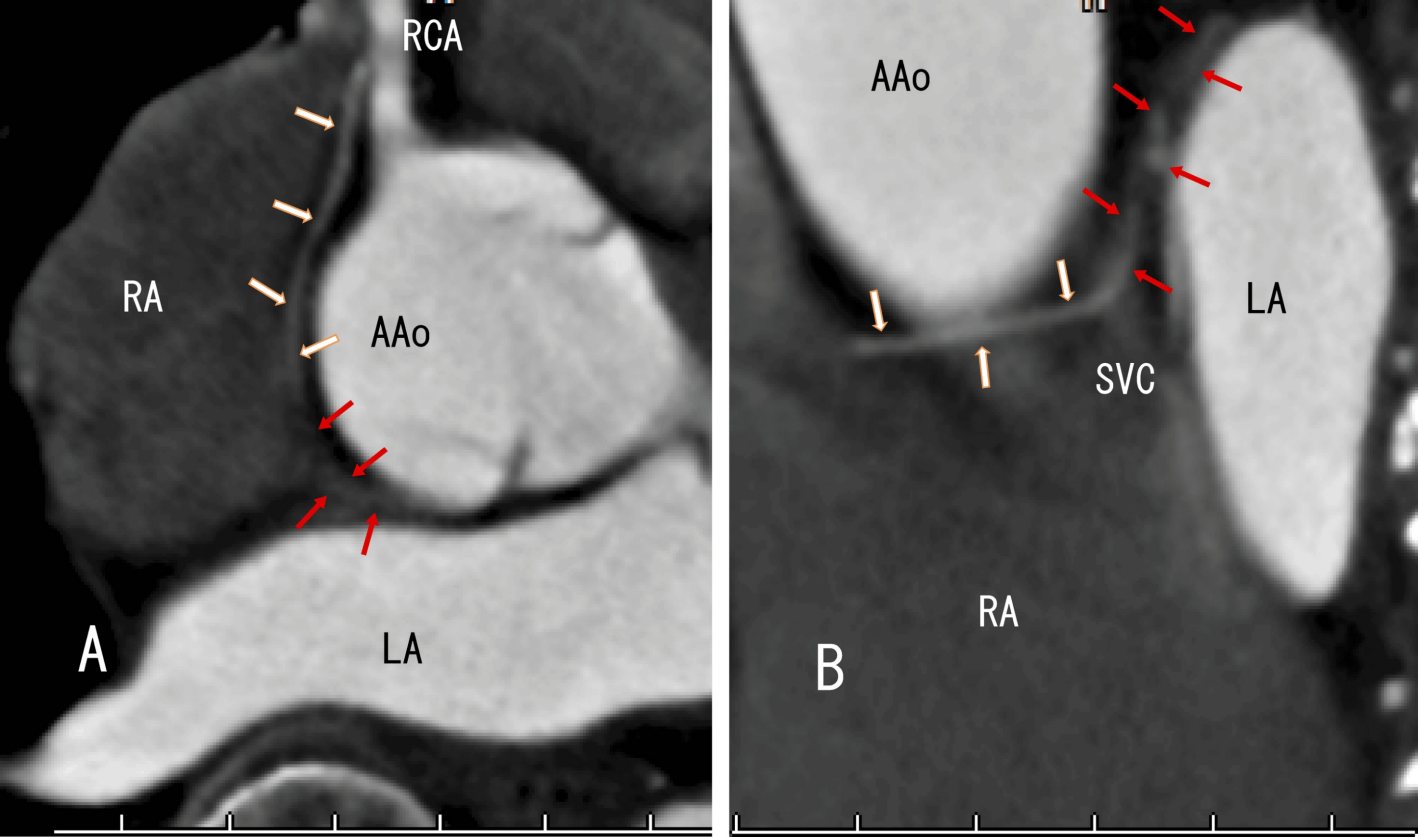
Enhanced CT images showing the AAo and LA. Enhanced CT (A: axial, B: sagittal) revealed an atypical RCA (white arrows) and continuous vague whitish areas (red arrows), suggesting an arterial blood supply. CT: computed tomography; AAo: ascending aorta; LA: left atrium; RCA: right coronary artery; RA: right atrium; SVC: superior vena cava

Cardiac CT clearly revealed an atypical artery from the RCA (Figure 2, white arrows), which connected vague whitish areas among the LA, AAo, and SVC (Figure 2, red arrows); however, cardiac CT did not reveal RUPV thrombi, linear white thrombi, or AAo thrombi, which are similar to left atrial thrombi that extend from PVTs.

**Figure 3:**
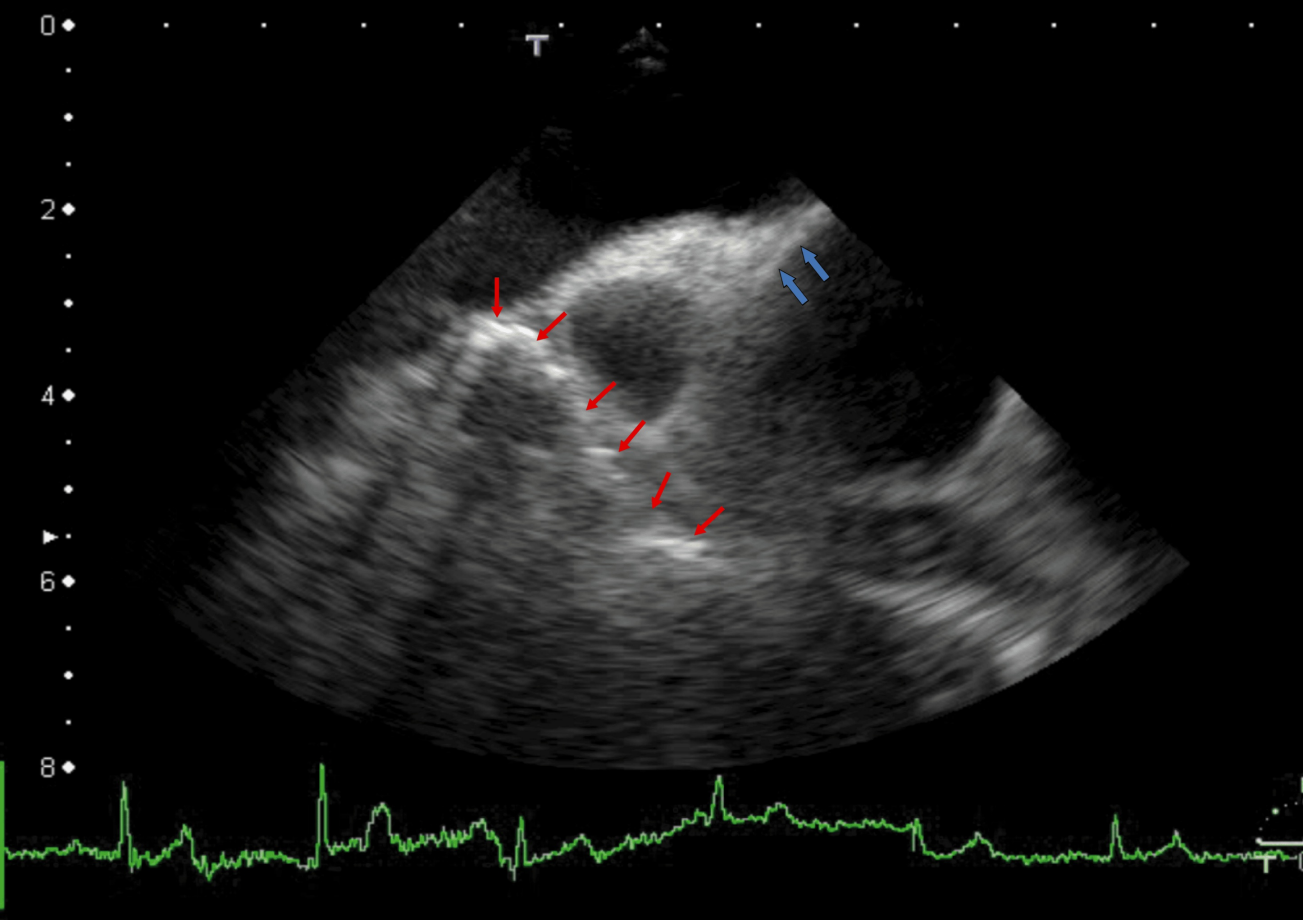
TEE image showing resolution of some linear vessel thrombi after six months of standard-dose apixaban treatment. After six months of standard-dose apixaban treatment, TEE revealed that some linear vessel thrombi had resolved (red arrows). Only right-sided thrombi exhibited high-echo-intensity shadows. Some areas of the AAo wall showed a normal structure (blue arrows). The cleft in the low-echo-intensity areas had disappeared. TEE: transesophageal echocardiography; AAo: ascending aorta

I treated the patient with apixaban (5 mg, twice daily) for six months. A normal AAo wall was observed in some areas (Figure 3, blue arrows). Most AAo thrombi did not resolve; however, the linear white thrombi partially resolved, and the cleft in the AAo wall disappeared (Figure 3 and Video 2). Cardiac CT images were similar to those in Figure 2. FEV1.0% increased to 69.3%.

**Video 2:** TEE video showing resolution of some linear high-echo-intensity thrombi after six months of apixaban treatment. After six months of treatment with a standard dose of apixaban, TEE revealed that some linear high-echo-intensity thrombi had partially resolved and did not move with heartbeats. The angle of this video was the same as that in Figure 3. TEE: transesophageal echocardiography

**Figure 4:**
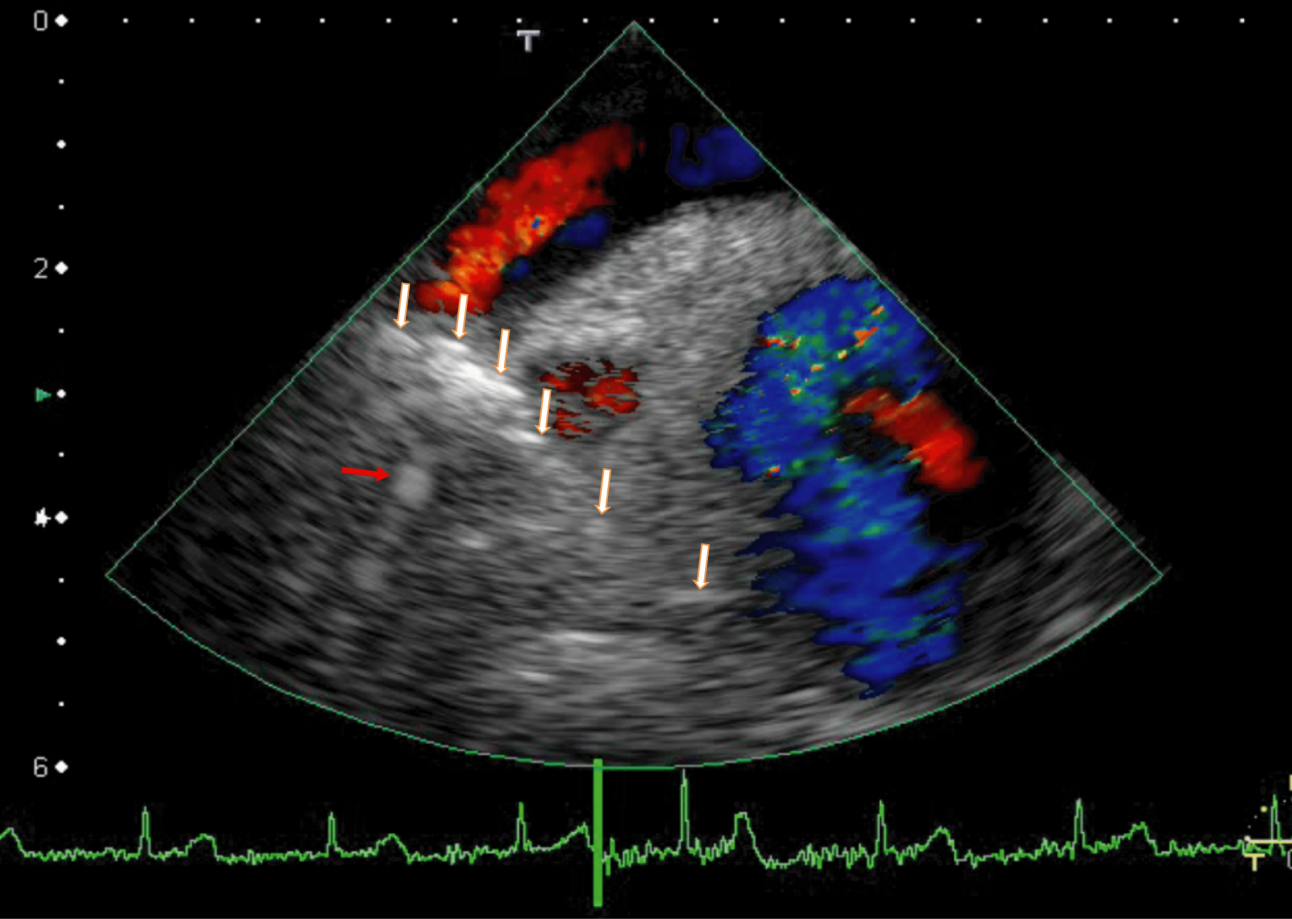
TEE image showing resolution of some vessel thrombi after one year of half-dose apixaban treatment. After one year of half-dose apixaban treatment, TEE revealed that the left-sided vessel thrombi had resolved and did not move with heartbeats. The right-sided thrombi exhibited high-intensity echoes (white arrows). The red arrow indicates a finding with white shadows near the RUPV thrombi. TEE: transesophageal echocardiography; RUPV: right upper pulmonary vein

For the next year, I treated the patient with decreased dosages of apixaban (2.5 mg, twice daily) to prevent side effects. The AAo thrombi subsequently did not change, and the left side of the connecting thrombi between the RUPV thrombi and AAo thrombi disappeared (Figure 4 and Video 3). Cardiac CT images were similar to those in Figure 2. Furthermore, FEV1.0% increased and was within the normal range (72.0%).

**Video 3:** TEE video showing partial resolution of some linear high-echo-intensity thrombi after one year of half-dose apixaban treatment. TEE was similar to that in Video 2. The white shadows of the linear thrombi had partially disappeared and showed slight movement with heartbeats. The angle of this video was the same as that in Figure 4. TEE: transesophageal echocardiography

The main points of treatment are summarized in Table 1, and the initial laboratory results are summarized in Table 2.

**Table 1:** Main points of treatment. DOACs: direct oral anticoagulants; AAo: ascending aorta; BNP: brain natriuretic peptide; FEV1.0%: percentage forced expiratory volume in one second

Item	Start	6 months	18 months	Normal range
DOACs	-	Apixaban (5 mg, twice daily)	Apixaban (2.5 mg, twice daily)	-
AAo thrombi (cm)	3 × 0.8	2.5 × 1.5	2.5 × 1.0	-
The mass (cm)	1.0 × 0.8	1.0 × 0.8	1 × 0.3	-
Line-like white thrombi (cm)	6 × 1 white + 2 shadows do not move with heartbeats	6 × 1 less white + 1 shadow does not move with heartbeats	3 × 1 less white + 1 less shadow move with heartbeats	-
The cleft (cm)	0.6 × 0.2	Disappeared	Disappeared	-
Creatinine (mg/dL)	0.77	0.75	0.79	0.47-0.79
BNP (pg/mL)	2.9	8	8	<18.4
D-dimer (μg/mL)	0.7	0.5	0.5	<1.0
FEV1.0%	60.2%	69.3%	72.0%	>70.0%

**Table 2:** Initial laboratory results. Hb: hemoglobin; Plt: platelet; BUN: blood urea nitrogen; TG: triglyceride; HDL-C: high-density-lipoprotein cholesterol; LDL-C: low-density-lipoprotein cholesterol; CRP: C-reactive protein; FT4: free thyroxine (3,5,3′,5′-tetraiodo-L-thyronine); FT3: free 3,3′,5′-triiodo-L-thyronine; TSH: thyroxine-stimulating hormone; ApoA1: apolipoprotein A1; ApoA2: apolipoprotein A2; ApoB: apolipoprotein B; ApoC2: apolipoprotein C2; ApoC3: apolipoprotein C3; ApoE: apolipoprotein E

Item	Normal range	Results
Hb (g/dL)	14-18	14
Plt (× 10^4^/μL)	15-40	59.6
Fibrinogen (mg/dL)	150-400	322
BUN (mg/dL)	8-20	14.5
TG (mg/dL)	35-150	149
HDL-C (mg/dL)	40-100	42
LDL-C (mg/dL)	70-140	115
CRP (mg/dL)	<0.30	0.52
FT4 (ng/dL)	0.93-1.75	0.99
FT3 (pg/mL)	2.5-3.5	2.6
TSH (μU/mL)	0.65-5.55	1.16
ApoA1 (mg/dL)	126-165	139
ApoA2 (mg/dL)	24.6-33.3	31
ApoB (mg/dL)	66-101	91
ApoC2 (mg/dL)	1.5-3.8	5.7
ApoC3 (mg/dL)	5.4-9.0	9.8
ApoE (mg/dL)	2.8-4.6	3.4

## Discussion

AAo thrombi with medial degenerative changes have been reported to cause AMI without obstruction of the right or left coronary artery [10,11,13]. AAo thrombi were detected by postmortem examination, where it was noted that the cleft and platelet fibrin thrombi in this cleft on the thrombogenic surface were the nidus of thrombus formation [13]. Kalangos et al. reported that thrombi in the distal AAo were detected by enhanced CT or transthoracic echocardiography (TTE) in four of eight patients; however, thrombi in the proximal AAo (approximately 1.5-2 cm above the aortic valve) were detected by TEE or autopsy but not by enhanced CT in four patients [12]. Importantly, proximal AAo thrombi were detected not by enhanced CT but by TEE, suggesting that the characteristics of proximal AAo thrombi differ from those of distal AAo thrombi. These reported AAo thrombi were present for a long time; however, they did not contain calcifications. This is consistent with the present case, in which the AAo thrombi contained no calcifications. Enhanced CT was unable to detect proximal AAo thrombi, RUPV thrombi, or linear white thrombi.

In the present case, the PAT was supplied by an atypical artery from the RCA; however, it is traditionally thought that the PAT is not supplied by coronary arteries such as the RCA. Recent research on EAT and PAT has increased, making these findings important. I previously reported that an atypical artery from the RCA supplied a left atrial diverticulum [5]; the route of the atypical artery from the RCA was similar to that in the present case.

Apixaban treatment led to the resolution of the AAo wall abnormalities on follow-up TEE. Apixaban promoted a normal AAo wall and resulted in the disappearance of the cleft in the low-echogenic area on the AAo wall, as determined by TEE. The area of low echogenicity might represent plaque in the atherosclerotic region. An abnormal AAo wall, characterized by the area of low echogenicity and the cleft, was present between the SVC and the AAo, with AAo thrombi attached to the AAo wall. The area supplied by the atypical artery was near the abnormal AAo wall (approximately 1-3 cm); in particular, the posterior side of the abnormal AAo wall was close to the atypical artery supply region (approximately 1 cm).

There are approximately 3.2 billion cardiac myocytes in the heart, with turnover rates of these cells being less than 1% per year [16]. If the turnover rate is 0.1% per year, then about 9,000 cardiac myocytes turn over daily. CD34-positive cells have been detected around the attachment areas of the AAo and thrombus [11]. Retrieved thrombi contained CD34-positive cells, and high-mobility group box 1 expression was also detected in these thrombi [17]. CD34-positive cells exhibit excitation-contraction pairing traits similar to those of cardiac myocytes [18]. However, the relationship between these CD34-positive cells and cardiac myocytes remains unclear. While cell sheet therapy can cure severe heart failure in some cases, it cannot do so in others [19,20]. Clarifying the mechanisms underlying these AAo wall abnormalities, especially the role of CD34-positive cells, may lead to a deeper understanding of cell sheet therapy mechanisms and improve its efficacy; nonetheless, more studies are needed.

Limitations

This report describes only one case; therefore, strong conclusions cannot be drawn. Changes in the AAo wall were observed only using TEE, and the changes in the cells and thrombi could not be precisely clarified; thus, microscopic and genomic studies are needed.

## Conclusions

This case suggested the presence of a possible atypical arterial branch from the RCA toward the PAT near an abnormal proximal AAo wall. Follow-up TEE revealed partial resolution of the thrombi and disappearance of the cleft after apixaban therapy. These findings require confirmation in additional cases.
